# Abscess formation of a spherical-shape duplication in the splenic flexure of the colon: case report and review of the literature

**DOI:** 10.1186/1757-1626-2-158

**Published:** 2009-10-20

**Authors:** Christos Limas, Chrisostomos Soultanidis, Michail A Kirmanidis, Christina Tsigalou, Olga Tsirogianni

**Affiliations:** 1 Paediatric Surgery Department, University General Hospital of Alexandroupolis, Dragana, 68100 Alexandroupolis, Greece

## Abstract

Gastrointestinal tract duplications are rare congenital malformations that may occur anywhere in the alimentary tract from the mouth to the anus, and vary greatly in presentation, size, location, and especially in symptoms. We present a case of an infected spherical colonic duplication, in a 20-day-old baby, located at the splenic flexure of the colon. The prominent symptom was acute abdomen, accompanied by bilious vomiting, intestinal obstruction, and high fever. We present this case, due to atypical clinical presentation and the inability of the imaging modality to establish the diagnosis preoperatively.

## Introduction

Gastrointestinal tract duplication (GTD) is defined as a structure which shares a common muscular wall and blood supply with the adjacent portion of the gut. The epithelial lining of the duplication resembles a part of the gastrointestinal or respiratory tract [1]. They are rare congenital malformations with various, potentially dangerous manifestations and are classified according to their location, shape and the presence of ectopic tissue within these. GTDs occur in about one per 18.000 live births, (one in 5.000 neonatal autopsies). Any part of the alimentary tract from the mouth to the anus can be affected. The ileum and ileocecal valve are the most common locations following by duodenum, stomach, jejunum, colon, and rectum. The shape-cystic duplications are the most common (up to 80%), and ectopic tissue within the duplication is encountered in 1/3 of the cases, (gastric mucosa up to 50%). Most of GTDs are detected in the first two years of life or antenatally [2-4]. They typically present as abdominal masses, with signs and symptoms of acute intestinal obstruction, bleeding or perforation. The early diagnosis allows prompt treatment with fewer complications.

## Case report

**Figure 1:**
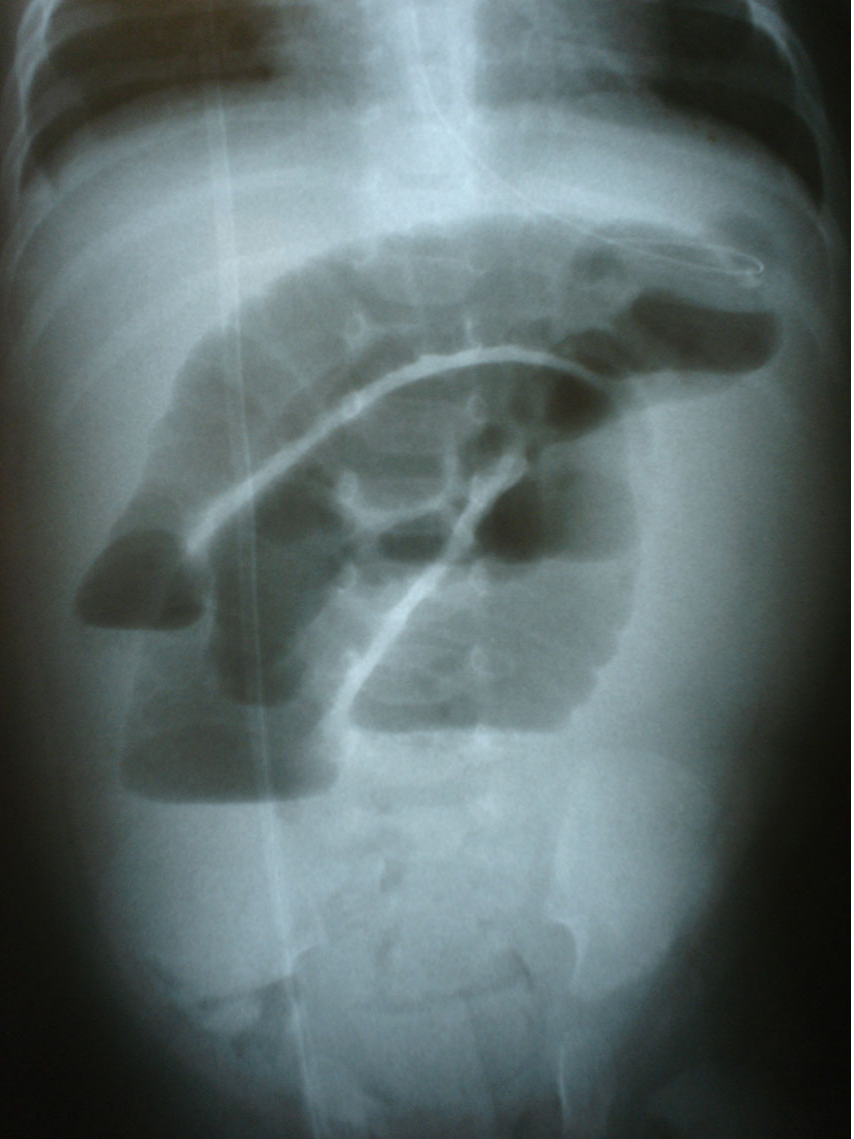
**Intestinal obstruction with air-fluid levels, distended and oedematous intestinal loops is showed in the upright x-ray**.

A 20-day-old male baby was admitted to the Department of Pediatric Surgery with a 24-hour history of abdominal pain and distension accompanied by bilious vomiting. His body temperature was 39.8°C, pulse 150 beats/min and blood pressure 90/45 mmHg. The physical examination revealed an extremely tender and massively distended abdomen with no peristalsis. A nasogastric tube that was inserted, produced a large amount of bilious fluid. Laboratory investigation revealed blood cell count 17,800/mm^3^, erythrocyte sedimentation rate 35 mm/h, whereas all other findings were normal. An upright plain abdominal radiograph revealed intestinal obstruction with air-fluid levels and massively distended and oedematous intestinal loops in the upper abdomen (Figure 1). Ultrasonography examination did not disclose the cause of the ileus. As the patient's clinical status was rapidly aggravated an immediate surgical exploration was necessitated.

At operation an inflamed mass was found in upper left quadrant, in which the splenic flexure, the spleen, the greater omentum and loops of the small intestine participated. Careful dissection of the adhesions, between the greater omentum and the intestinal loops, revealed a small amount of pus and an inflamed spherical mass (4 × 4 cm in size). This mass was full of pus and adherent to the antimesenteric border of the splenic flexure of the colon and shared a common muscle wall with it (2 × 2 cm in dimension). The spherical mass was isolated from the colon and removed easily. Examination of the colon and the mass did not reveal any communication between them. The muscle coat of the colon was simply sutured. The histopathological examination of the excised specimen showed histological patterns resembling the colon, resulting in the final diagnosis of colonic obstruction due to an inflamed and purulent non-communicating spherical duplication in the splenic flexure of the colon.

The postoperative course was uneventful and the baby was discharged on the 6^th ^postoperative day.

## Discussion

The first report of a GTD was published by Galder in 1733, and Ladd was the first that introduced the term "duplication of the alimentary tract" in 1937. Gross, in 1950s, classified the duplications into four variations according to the shape: tubular, double-barrelled, cystic and spherical. Tubular structures branch out of the intestine and extents for some distance between the mesenteric leaves. The double-barrelled duplications communicate with the intestinal lumen at one or both ends. The cystic structures are attached by a thick mesenteric stalk only and are lying free in the peritoneal cavity. The spherical duplications, most common of all, are contiguous with some part of the bowel, particularly along the ileum [5]. According to arterial supply they are also classified into two types: parallel and intra-mesenteric. In the first type the parallel vessels can be separated, whereas the second type implies a resection of the cyst without the adjacent bowel as the duplication can be enucleated by ligation of the short branches, keeping the straight vessels of the intestinal segment intact [4]. Current nomenclature relies on the anatomic location in relation to the normal intestine and not on the histological features of the mucosal lining [6]. The aetiology is not known although several theories have been postulated, such as the split notochord, the failure in the regression of embryonic diverticula, the external compression of adjacent loops, the errors in the epithelial recanalization, and intrauterine environmental factors [4].

Approximately 75-85% of duplications are located within the abdominal cavity, and the remaining are intrathoracic (20%) or thoracoabdominal (5%) [3]. Multiple duplications are also encountered in 5-15% [7]. They may be cystic (75-80%), saccular or tubular in shape, with or without communication with the adjacent intestine. They are most often located on the mesenteric side of the native gastrointestinal tract structure [8-10]. The small intestine is the most frequent site involved (60%), especially the ileum, followed by mediastinal duplications (18%), whereas stomach (7%), duodenum (6%), colon (15%), rectum (4%), and neck (1%) are relatively rare [3-5,8-12]. In our case the spherical mass was found in the antimesenteric side of the colon, which represents an uncommon location. Heterotopic mucosa, mostly gastric type, is contained in 33% of the cases, although more than one type in the same duplication is encountered in 10% of the cases [7]. The male to female ratio is 5/4 [7]. Other congenital abnormalities such as vertebral, urogenital, intestinal atresia or stenosis, omphalocele, and malrotation associate duplications of the gastrointestinal tract in a percentage up to 25-40% [4,11,13].

Alimentary duplication should be considered in any pediatric patient suffering from unexplained and non-specific gastrointestinal symptoms with atypical clinical presentation, which makes a correct diagnosis difficult, and in many cases delayed for a long time. Approximately two thirds of all intestinal duplications are discovered within the first two years of life, with one third identified in the newborn period. However in some series the percentage of symptomatic patients in the neonatal period is higher (69%) [7]. Symptomatic GTDs present as abdominal masses that may accompanied by pain. Clinical manifestations are dependent primarily on location, shape, size, communication with the gastrointestinal tract, and the presence of heterotopic mucosa. Symptoms are produced when compression, intussusception or volvulus of the attached bowel causes intestinal obstruction [1]. The spectrum of symptoms includes intermittent vomiting, abdominal pain (50%), abdominal distension (30%), palpable mass (20%), peritoneal signs (13%), constipation (6%), bleeding (6%) and perforation, but there is no common clinical pattern of signs for identifying GTDs [5,8,13]. Bleeding may be observed despite the lower prevalence of ectopic gastric mucosa in colon duplications. Obstruction and intermittent vomiting were the most frequent symptoms in some series [4,9]. In general, the most prominent symptoms are related to intestinal obstruction and peptic ulceration caused by ectopic gastric mucosa. Symptoms mainly occur in older patients, while neonates or very young children rarely present with symptomatic intestinal duplications. Newborns may present with volvulus or acute intestinal obstruction [4], whereas in neonates and infants vomiting and distension are the most common presentations. Volvulus occurs in 24% of neonates and intussusception is identified in 11%. In older children and adolescents, pain and vomiting are the most common presentation [3]. In many cases preoperative diagnosis cannot be made, because of the wide spectrum of the signs and symptoms [7]. In the literature abscess formation of a GTD has not been described yet, as far as we know.

Ultrasonography is the imaging modality of choice for the detection and diagnosis of duplication cysts, as it will identify a three-layered image, unless complications such as haemorrhage and infection have supervened [1,9]. Approximately 30 to 50% of the intestinal duplications are diagnosed antenatally and the number is gradually increasing [2-4]. Plain thoracic and abdominal X-rays, as well as computed tomography of the chest and the abdomen are also used as diagnostic radiological methods [7]. However, gastric, duodenal, and rectal duplications can lead to a diagnostic dilemma [2]. In our patient the correct preoperative diagnosis was a subsidiary matter, as his general status rapidly deteriorated, and moreover ultrasonography did not disclose the cause of the ileum. In such cases diagnosis can be made in the theatre only and diagnostic imaging procedures must be left for not urgent cases.

Although GTDs considered benign lesions, if they are not treated properly they can have lethal consequences. Most of the authors believe that once the diagnosis is made, an elective surgical procedure should be performed to avoid complications and to perform the procedure in an optimal state of the patient, but few think that only symptomatic duplications should be surgically treated [2]. If incidentally encountered, duplications should be surgically addressed to avoid future complications. An elective surgical procedure should not be delayed once a definitive diagnosis has been made. Serious complications such as perforation or torsion, although rare, are difficult to be accurately diagnosed preoperatively and require urgent surgical intervention [2,12,14]. The recommended surgical procedure is excision of the duplication, and recent studies show that it is possible to resect them without compromising the adjacent bowel vascularization. Other procedures like marsupialization with mucosectomy, internal drainage, and Wrenn's procedure are also recommended when necessary [2]. In the management of the extensive lesions, it must be kept in mind that duplications are benign and should be treated in a conservative way without jeopardizing vital structures [5]. The malignant potential risk, if a portion of a duplication cyst is left in place, even theoretical, it may occur.

## Consent

Written informed consent was received from the patient's parents for publication of this case report and accompanying images. A copy of the written consent is available for review by the Editor-in-Chief of this journal.

## Competing interests

The authors declare that they have no competing interests.

## Authors' contributions

CL performed the exploration and wrote the paper. CS helped in the exploration and in the writing of the paper. MAK helped in the exploration and reviewed the literature. CT reviewed the literature. OT, helped in the review of the literature and the writing. All authors read and approved the final manuscript.
